# Neurocognitive challenges Post-COVID: current perspectives and future solutions

**DOI:** 10.1007/s00406-025-01962-9

**Published:** 2025-01-24

**Authors:** Tarek Jebrini, Hans Christian Stubbe, Michael Ruzicka, Kristina Adorjan

**Affiliations:** 1 Department of Psychiatry and Psychotherapy, LMU University Hospital, LMU Munich, Nußbaumstraße 7, 80336 München, Germany; 2 Department of Medicine II, Hospital of the LMU Munich, 81377 Munich, Germany; 3 Department of Medicine III, LMU University Hospital, LMU Munich, Munich, Germany; 4 Institute of Psychiatric Phenomics and Genomics (IPPG), LMU University Hospital, LMU Munich, Munich, Germany; 5 University Hospital of Psychiatry and Psychotherapy, University of Bern, Bern, Switzerland

**Keywords:** Post COVID, Long COVID, COVID-19, Pandemic, Cognitive impairment

In the year 2019 the sudden appearance of the coronavirus disease in 2019 led to the most severe health crisis of our century so far, leading to up to 776 million confirmed infections and more than 7 million deaths around the globe [1].

Considering results of recent meta-analyses, a minimum of 10% of COVID-19 survivors have developed post-COVID conditions (PCC) symptomatology after an acute SARS-CoV-2 infection [2].

Recent research in the field of PCC has made substantial progress in understanding the multisystemic disease. Particularly regarding the understanding of associated pathophysiological changes, associated risk factors, and the disease progression, significant progress was made. Nevertheless, the broad variety and complexity of symptom clusters taken together with the overlap between PCC symptoms of psychosomatic and psychiatric syndromes continue to pose significant challenges in clinical diagnosis and treatment.

The current special issue of the European Archives of Psychiatry and Clinical Neuroscience focuses on PCC, presenting novel evidence across multiple research dimensions of PCC which implement various methodological approaches. This collection of articles provides in-depth insights into the latest findings and current literature concerning the diagnosis, treatment, and clinical features of PCC and associated psychiatric conditions. Furthermore, it contextualizes these findings by drawing comparisons with other related disorders and relevant methodological approaches. The variability in symptomatology is underlined by the various and diverse publications included.

A highlight of this special issue is the exploration of cognitive impairment (CI) symptomatology in PCC. In their qualitative systematic review, Espinoza and Martella emphasize the significant heterogeneity in the reported prevalence rates of CI, which depends on exploration methods, follow-up duration, and whether deficits are assessed through objective measures or self-reporting [3]. Their analysis includes a Swedish study by Wahlgren et al. published in the Lancet in 2022 involving 745 COVID-19 survivors, 185 of whom reported persistent symptoms. Among these patients, 48% self-reported cognitive deficits five months after discharge, while 37% were identified through neuropsychological assessment, and only 3% were documented by physicians [4]. This discrepancy illustrates the complexity and difficulty of assessing, measuring, and defining CI, its prevalence, the associated clinical implications, and the impact on quality of life.

Another notable study highlighted by Espinoza and Martella is a longitudinal analysis of 401 participants from the UK Biobank published by Monje & Iwasaki in 2022, offering insights into cognitive evaluation before and after SARS-CoV-2 infection. Test results revealed a significant decline in cognitive function from baseline pre-COVID-19 levels compared to 384 healthy controls [5].

Evidence regarding the persistence of CI in PCC remains elusive. Espinoza and Martella reference a prospective, multicenter observational cohort study from Belgium by Rousseau and colleagues, involving 695 COVID-19 survivors. The study showed partial improvement up to complete recovery from CI in most participants within one-year post-infection [6].

Ruggeri and colleagues focused on the prediction of cognitive impairment (CI) in PCC and aimed to address this research question. Contrary to existing literature, their clinical study found no significant associations between CI and respiratory distress, comorbidities, or depression. Instead, only anosmia emerged as a predictive factor for memory disorders and was linked to temporo-mesial cognitive dysfunction. The authors hypothesize that both anosmia and temporo-mesial cognitive dysfunction may result from a shared pathophysiological mechanism, potentially involving SARS-CoV-2 neuroinvasion of the limbic brain. They propose that the virus might travel to limbic brain regions through a retrograde axonal olfactory transcribial route and highlight shared regions and functional connections between olfactory and memory systems [7].

Another key topic in this special issue explores pharmacological prevention and treatment strategies for PCC, particularly concerning cognitive impairment. In a letter to the editor, Bonnet and Kuhn propose that neuropsychiatric symptoms of PCC, including depression, anxiety, fatigue, and cognitive dysfunction, may result from a premature but potentially reversible aging process of midbrain dopaminergic neurons. They suggest the use of antidepressants (ADs) as a preventive intervention strategy, based on their antidepressant, anxiolytic, anti-inflammatory, and antiviral properties [8].

Finsterer focuses on neuropathic pain in PCC [9], stating that around one-third of COVID-19 patients have been reported to develop neurological symptoms including neuropathic pain and paraesthesia lasting up to 12 weeks [9]. The author is stressing the paucity of research in this critical area and underlines the lack of neurophysiological understanding of the symptomatology. Finsterer emphasizes the urgent need for comprehensive diagnostic and treatment strategies regarding neuropathic pain in PCC and calls to action.

The study by Schwarz et al. is focusing on another research topic, but applying an approach, which could be relevant for a deeper understanding in PCC conditions as well.

Schwarz et al. investigate the effects of ChemSex on brain function with a methodologically elegant approach using EEG assessments to examine real-time neural correlates of impaired cognitive functions, such as decision-making processes. The paradigm used by Schwarz et al. could be used as an additional measure for CI in PCC [10].

In summary, this special issue underlines the importance of a multilevel perspective on PCC, integrating various multimethodological expertise to study PCC comprehensively. Linking current and future findings on PCC to prior research on related disorders is crucial for developing a deep understanding of the condition. However, diagnostic and treatment options remain insufficient and lack standardization, especially regarding the common symptomatology of CI. Clinical studies are urgently needed to improve diagnostic accuracy and treatment efficacy, enabling better and sufficient care for patients with PCC in the near future.
